# Supplementary material for: Alcohol-Supported Cu-Mediated 18F-Fluorination of Iodonium Salts under “Minimalist” Conditions
Source: Molecules. 2019 Sep 3;24(17):3197. doi: 10.3390/molecules24173197 (PMC6749259; doi:10.3390/molecules24173197)
Supplement: Supplementary file 1 [file molecules-24-03197-s001.pdf]

Supporting information to

Alcohol-supported Cu-mediated ^{18}F-fluorination of iodonium salts under “minimalist” conditions

Victoriya V. Orlovskaya [1,†], Daniel J. Modemann [2,†], Olga F. Kuznetsova [1], Olga S. Fedorova [1], Elizaveta A. Urusova [2,3], Niklas Kolks [2,3], Bernd Neumaier [2,3,4,*], Raisa N. Krasikova [1,5,*] and Boris D. Zlatopolskiy [2,3,4]

[1] N.P. Bechtereva Institute of the Human Brain, 197376 St.-Petersburg, Russia; vikaorl@list.ru (V.V.O.); kuznetsova@ihb.spb.ru (O.F.K.); fedorova@ihb.spb.ru (O.S.F.);

[2] Institute of Neuroscience and Medicine, INM-5: Nuclear Chemistry, Forschungszentrum Jülich GmbH, 52425 Jülich, Germany; daniel.modemann@med.uni-goettingen.de (D.J.M.); e.urusova@fz-juelich.de (E.A.U.); niklas.kolks@uk-koeln.de (N.K.); boris.zlatopolskiy@uk-koeln.de (B.D.Z.)

[3] Institute of Radiochemistry and Experimental Molecular Imaging, University Clinic Cologne, 50937 Cologne, Germany

[4] Max Planck Institute for Metabolism Research, 50931 Cologne, Germany;

[5] St.-Petersburg State University, 199034 St.-Petersburg, Russia

* Correspondence: b.neumaier@fz-juelich.de (B.N.); raisa_krasikova@inbox.ru (R.N.K.)

† Contributed equally

Molar activity calculation: The molar activity (GBq/µmol) was calculated by dividing the radioactivity of the ^{18}F-labeled product by the amount of the unlabeled tracer determined from the peak area in a UV-HPLC chromatograms (λ=254 nm). The solution of 4-[^{18}F]FPhe obtained after HPLC purification was concentrated under reduced pressure, the residue was dissolved in a small amount of the HPLC eluent. The resulting solution was completely injected into the HPLC system. The peak area was determined and the amount of 4-FPhe was calculated according to the calibration curve. The molar activity of 4-[^{18}F]FPhe (2.22 GBq) was determined to 207 GBq/µmol.

Calibration curve for 4-[^{18}F]FPhe:

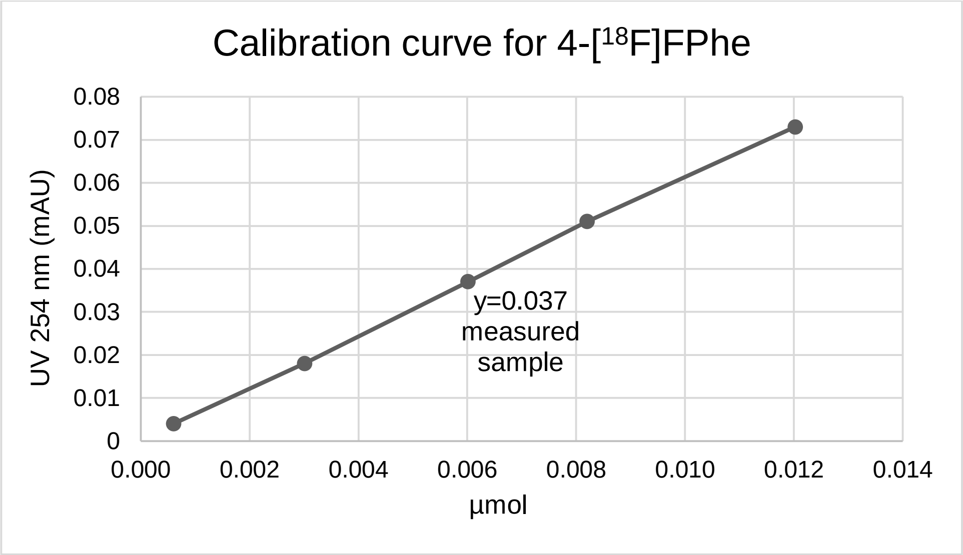

C, µmol	Peak area (mAU)
0.012	0.073
0.008	0.051
0.006	0.037
0.003	0.018
0.001	0.004

NMR and MS-spectra

(*S*,*S*)-Ni-BPB-3-IPhe (**14**):

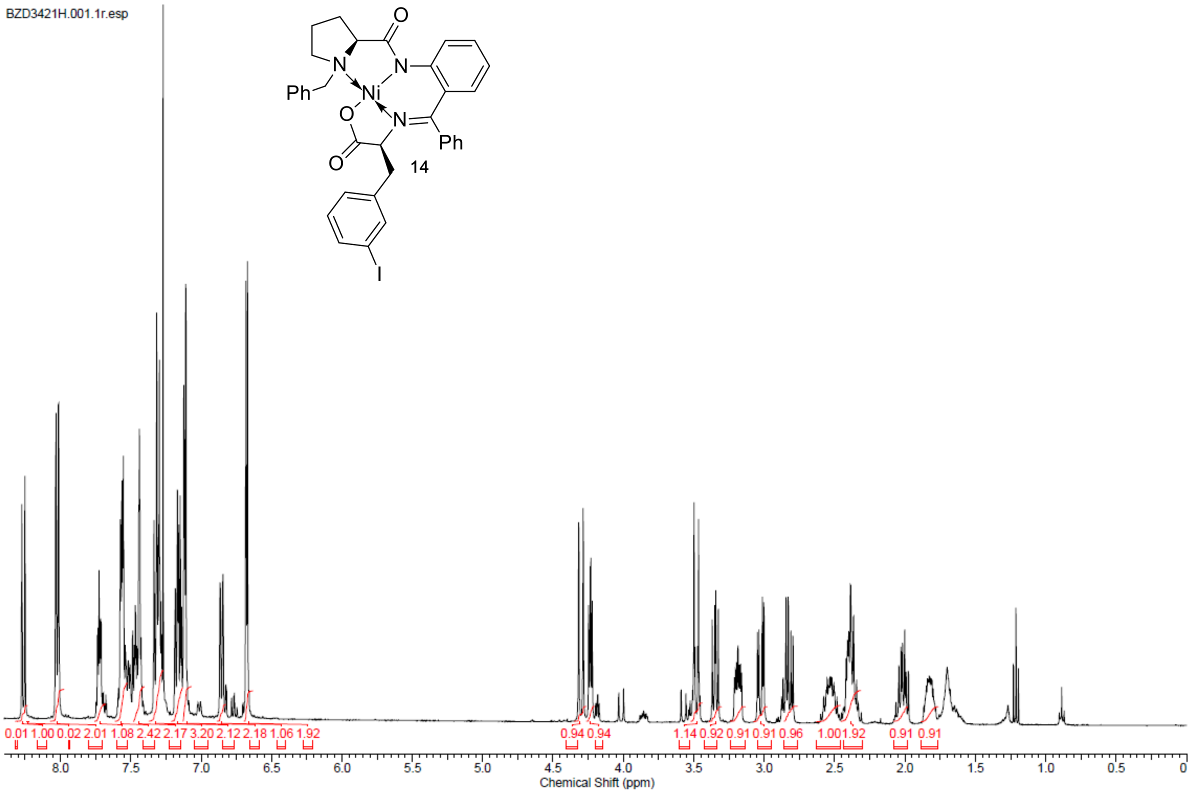

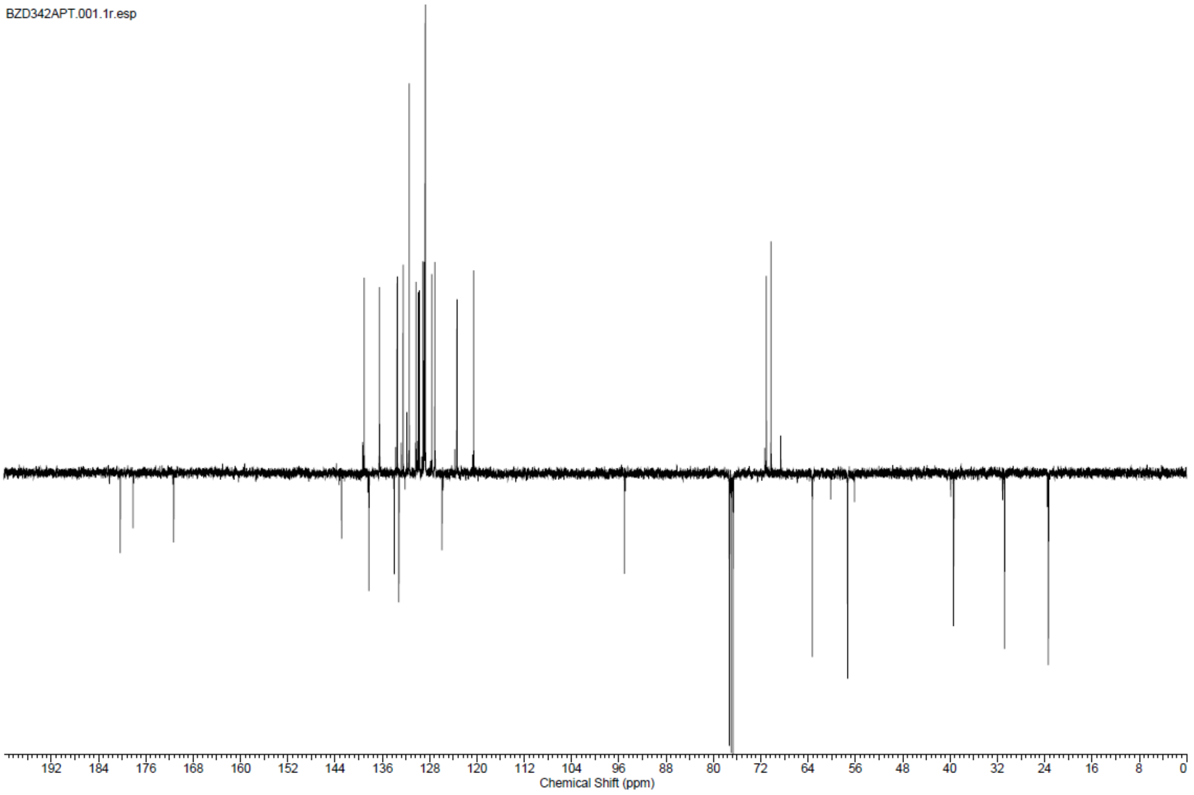

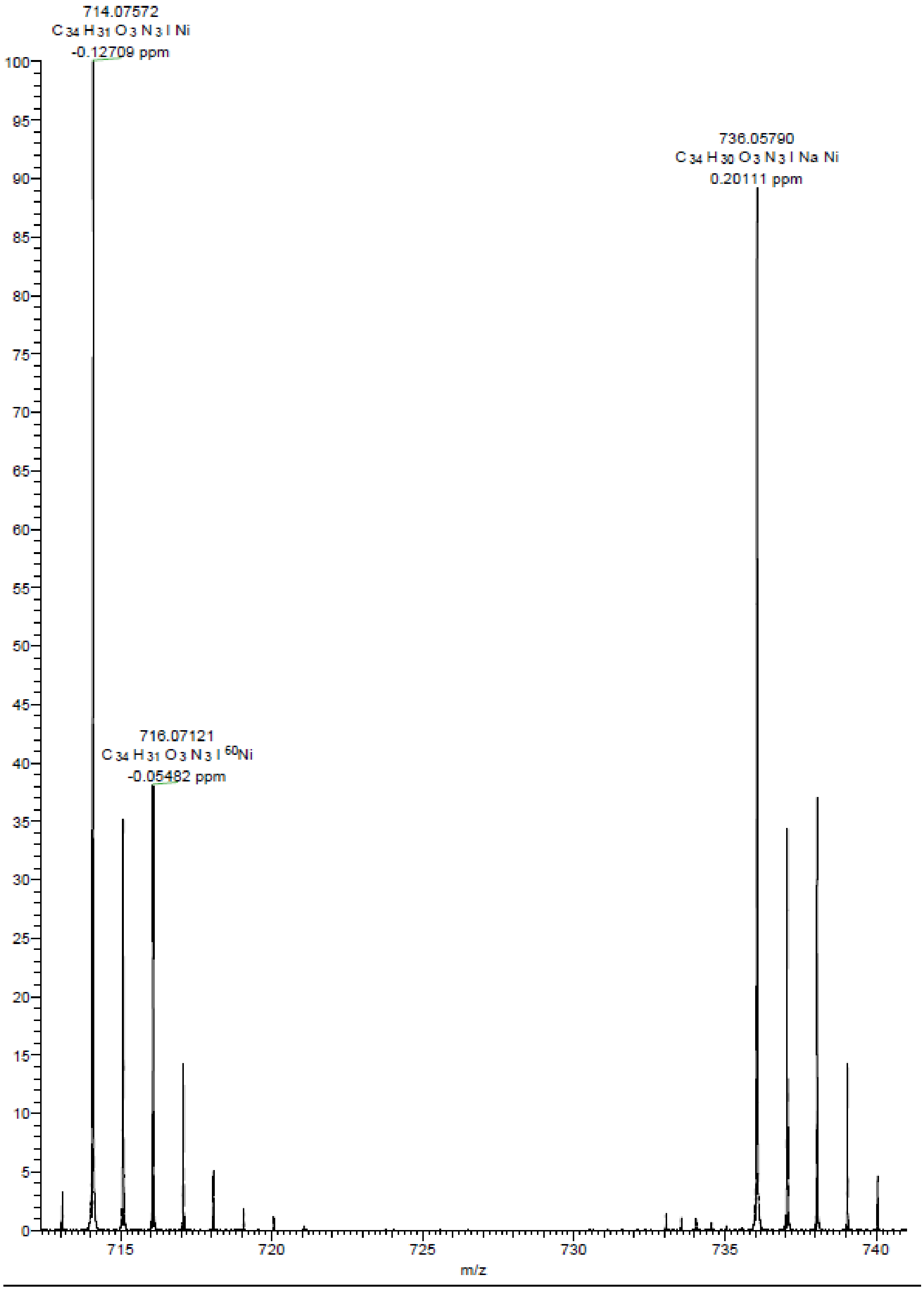
714.07572
C 34 H 31 O 3 N 3 I Ni
-0.12709 ppm
716.07121
C 34 H 31 O 3 N 3 I 60Ni
-0.05482 ppm
736.05790
C 34 H 30 O 3 N 3 I Na Ni
0.20111 ppm
m/z

Boc-3IPhe-OH:

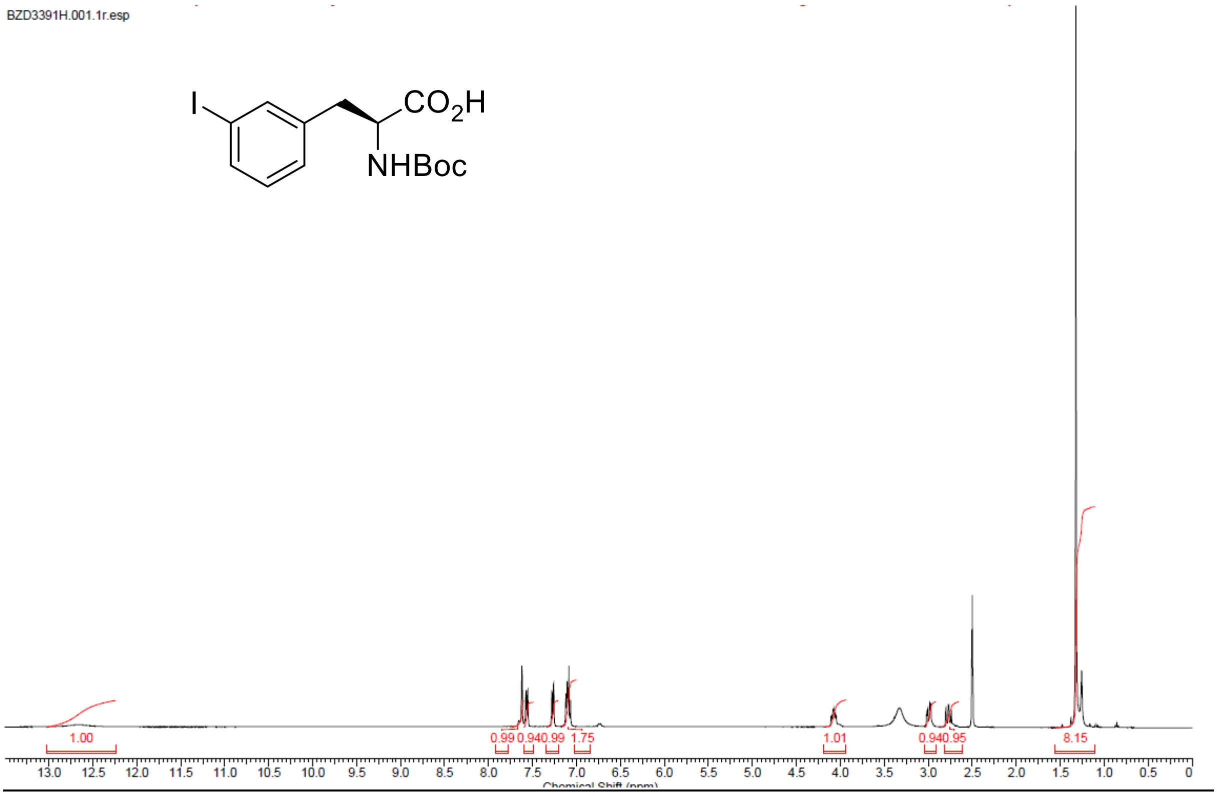

BZD3391H.001.1r.esp
CO2H
NHBoc
I
1.00
0.99
0.94
0.99
1.75
1.01
0.94
0.95
8.15
13.0 12.5 12.0 11.5 11.0 10.5 10.0 9.5 9.0 8.5 8.0 7.5 7.0 6.5 6.0 5.5 5.0 4.5 4.0 3.5 3.0 2.5 2.0 1.5 1.0 0.5 0

Boc-3-IPhe-O*t*Bu (**15**):

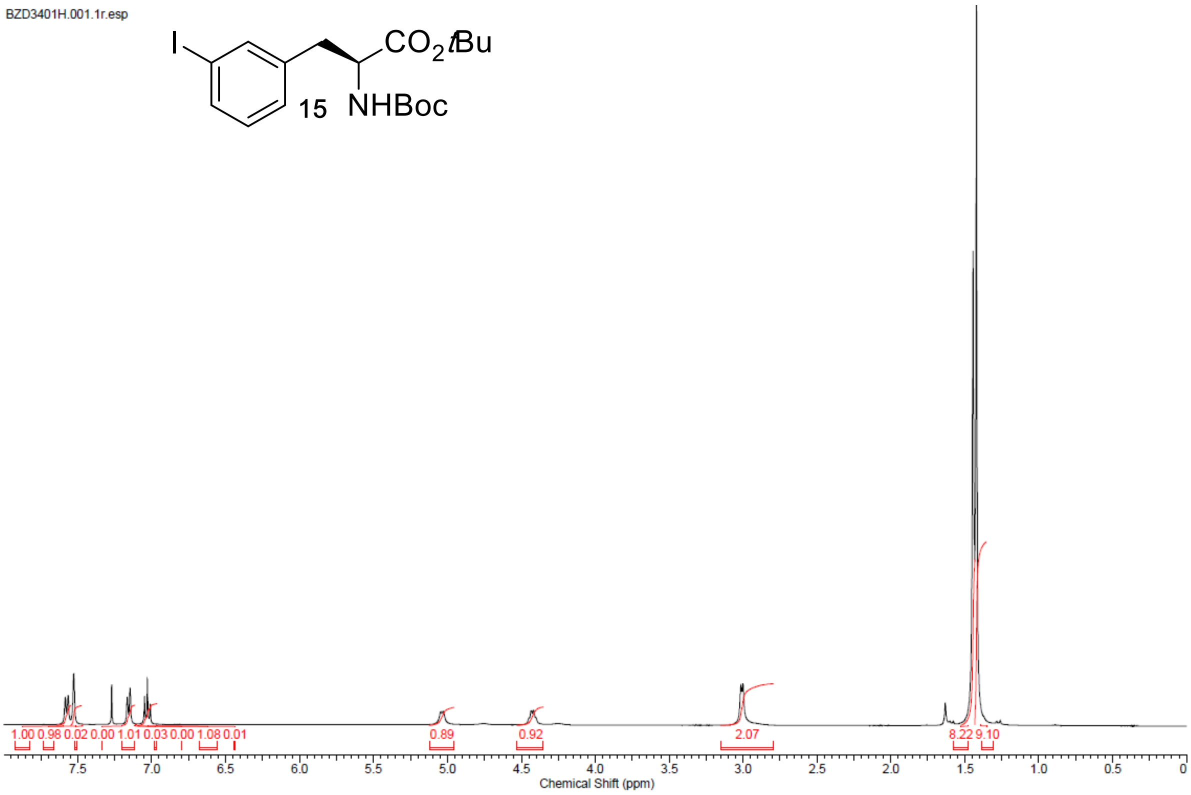

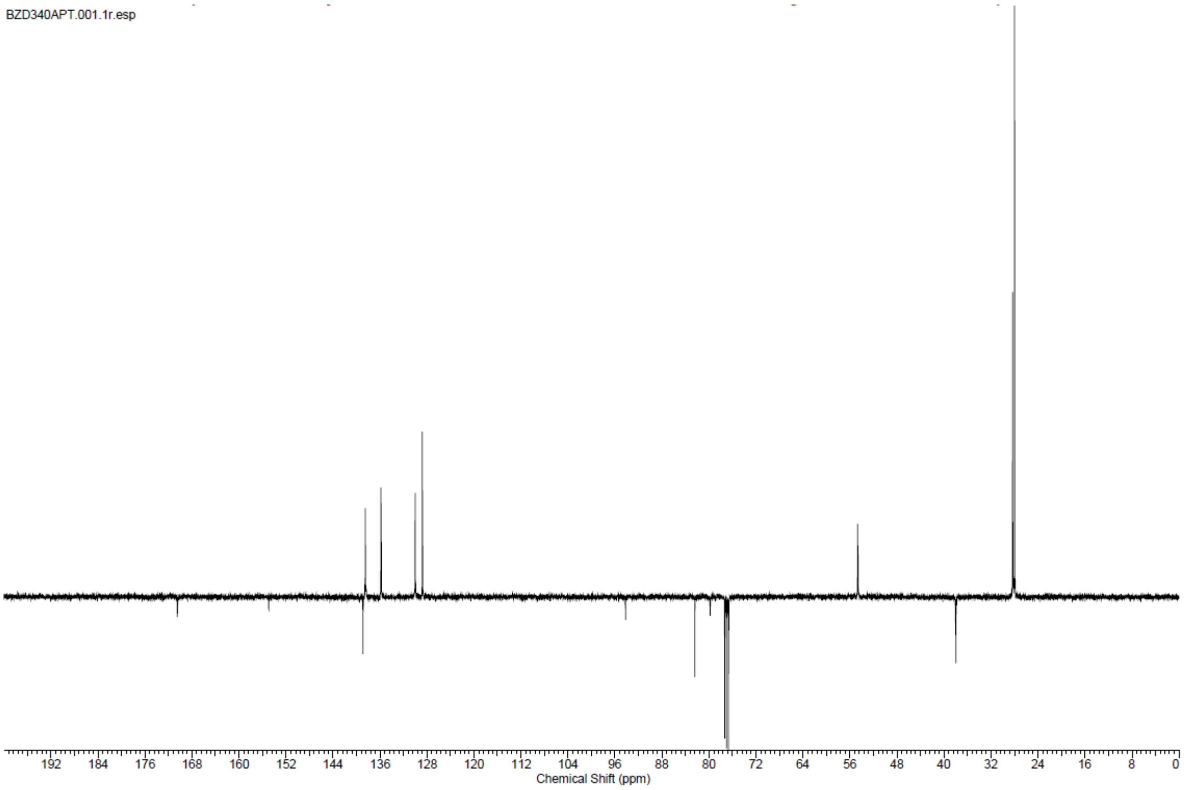

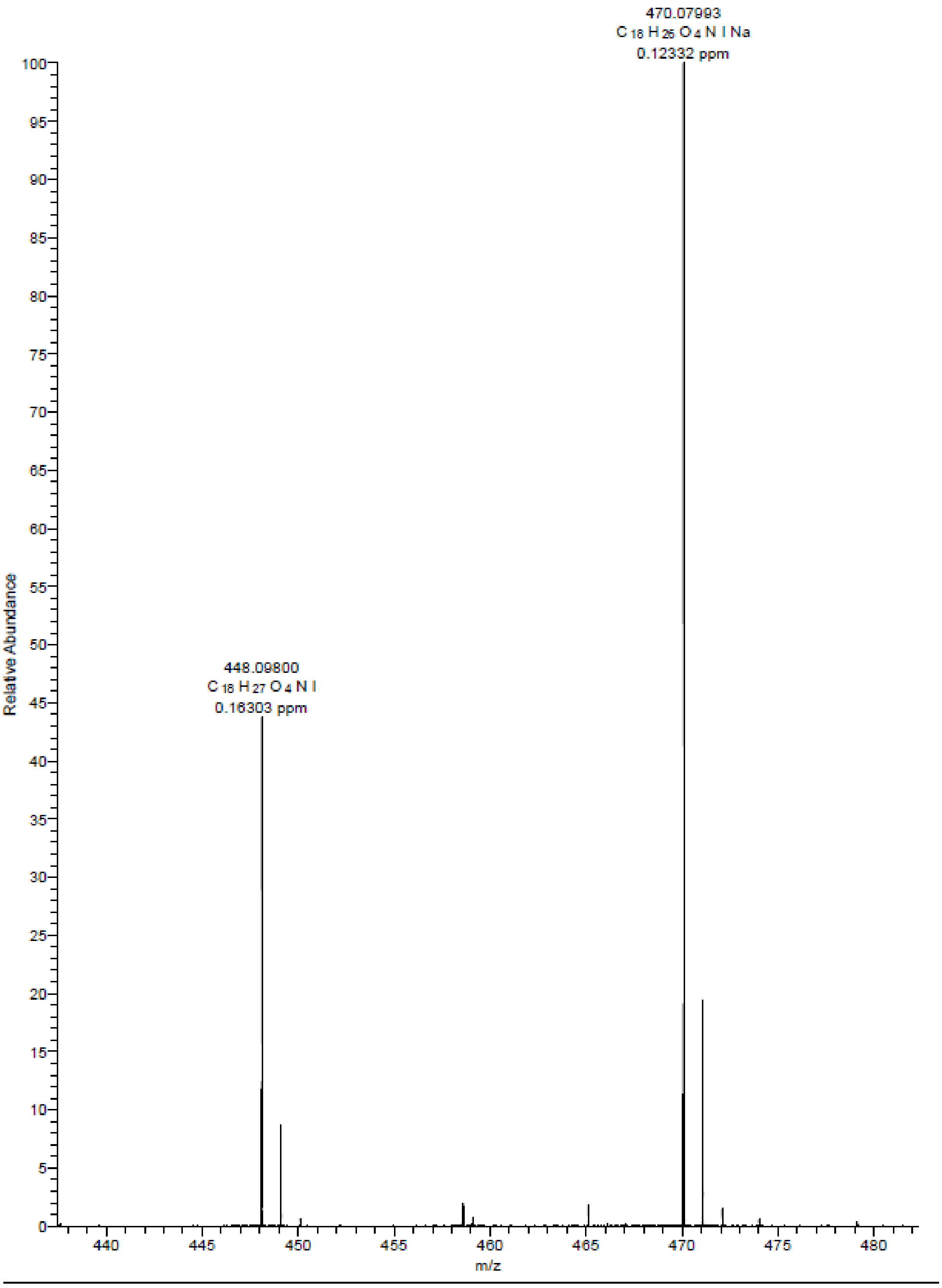
470.07993
C 18 H 26 O 4 N I Na
0.12332 ppm
448.09800
C 18 H 27 O 4 N I
0.16303 ppm
Relative Abundance
m/z

3-{2-[(2*S*)-2-{[(*tert*-Butoxy)carbonyl]amino}-3-(*tert*-butoxy)-3-oxopropyl]phenyl}(2,4,6-trimethylphenyl)iodonium tetrafluoroborate (**12**):

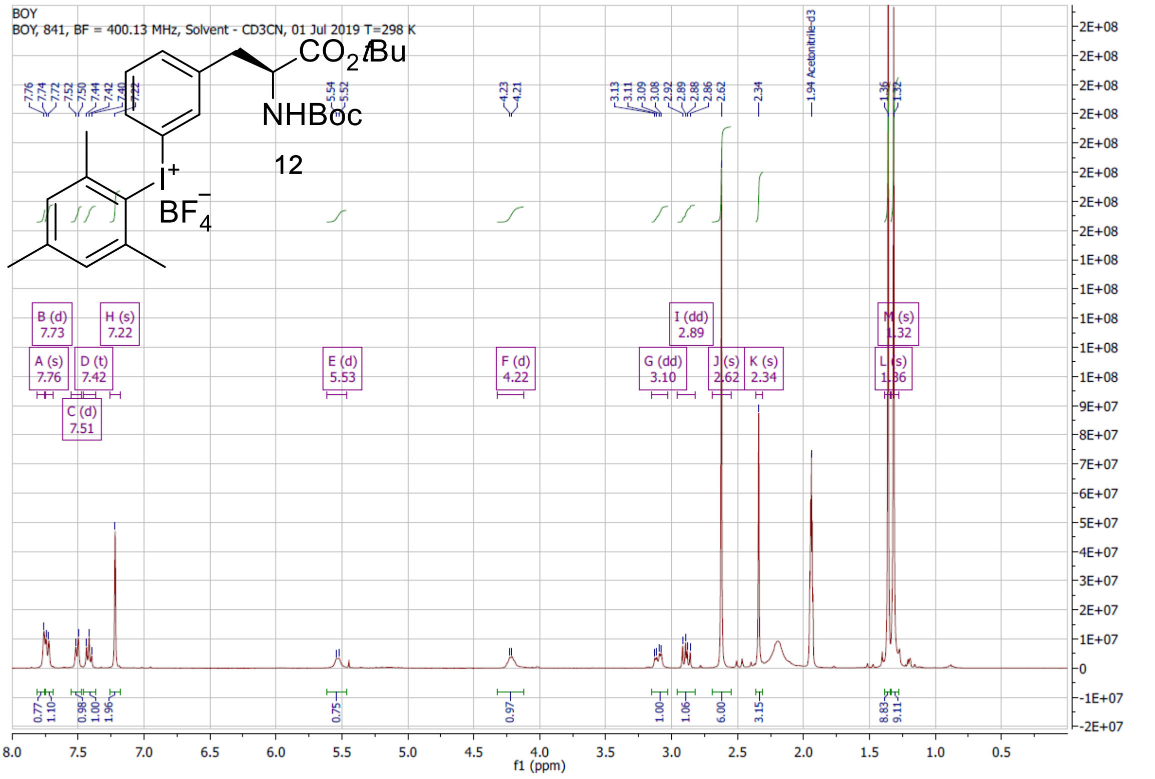

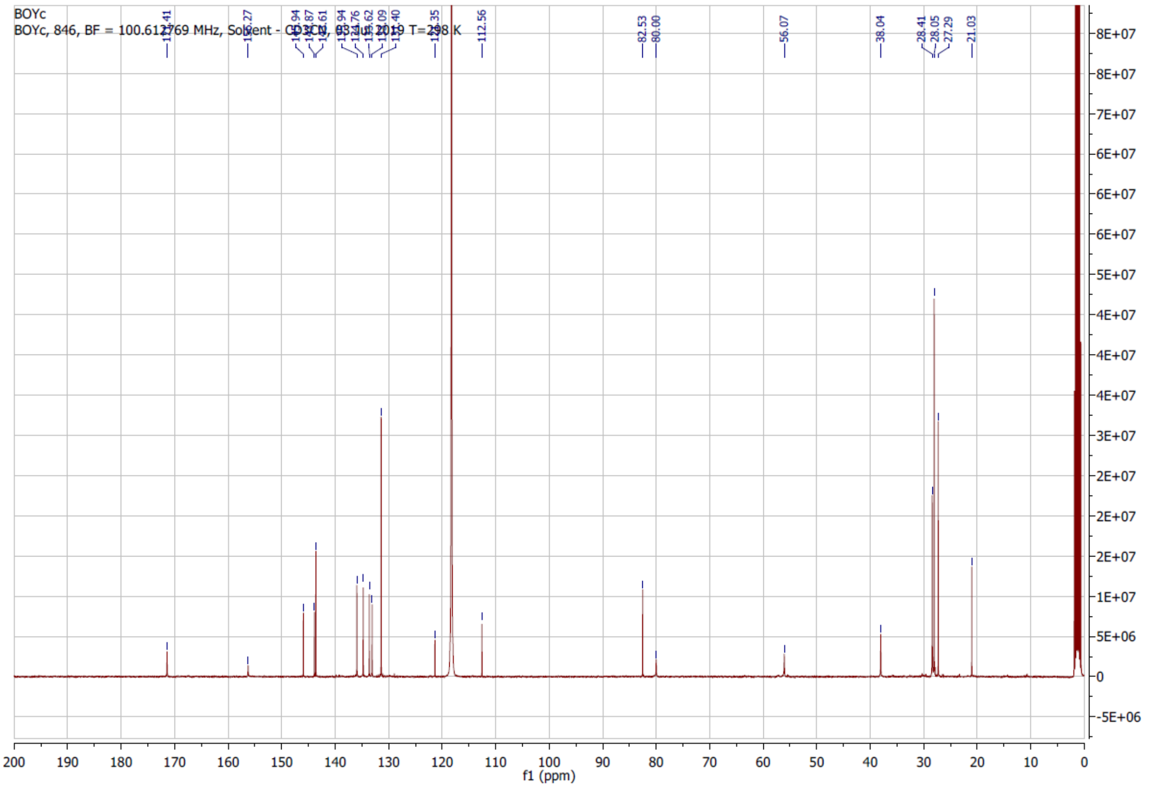

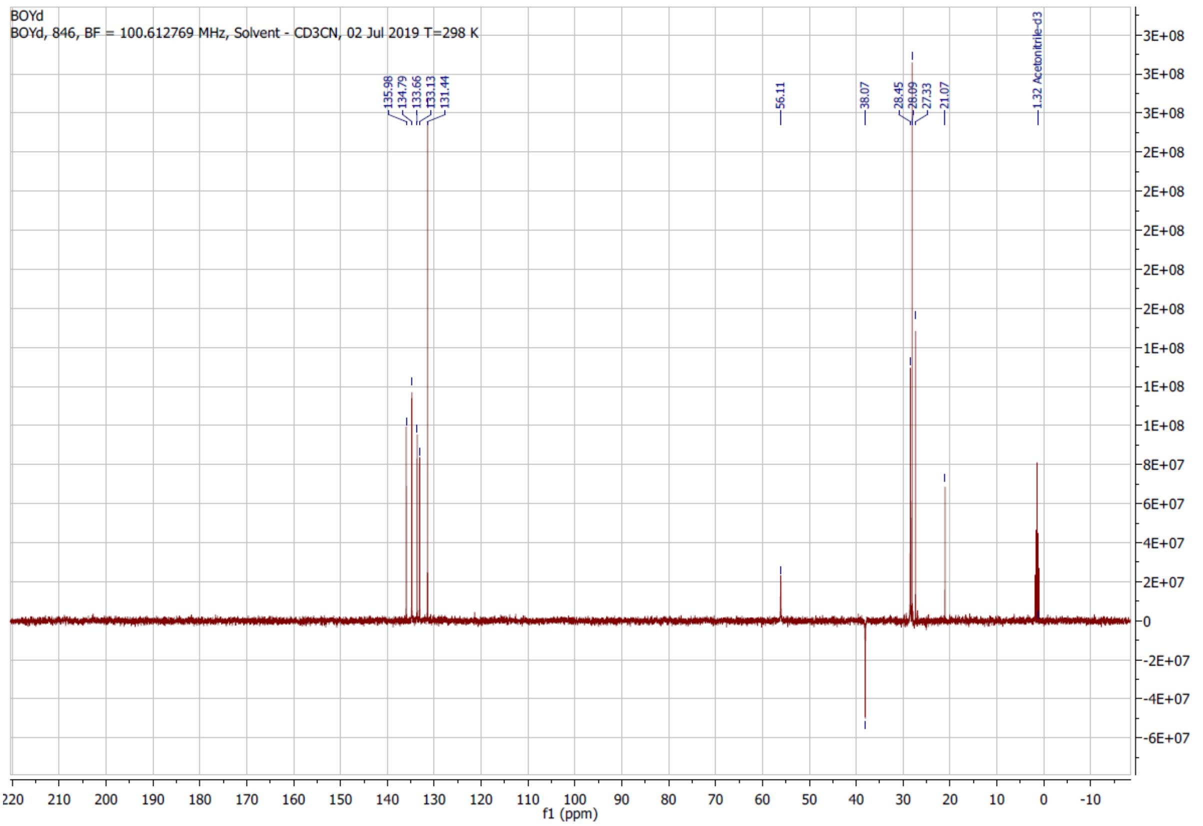
BOYd
BOYd, 846, BF = 100.612769 MHz, Solvent - CD3CN, 02 Jul 2019 T=298 K
135.98
134.79
133.66
133.13
131.44
56.11
38.07
28.45
28.09
27.33
21.07
1.32 Acetonitrile-d3
f1 (ppm)

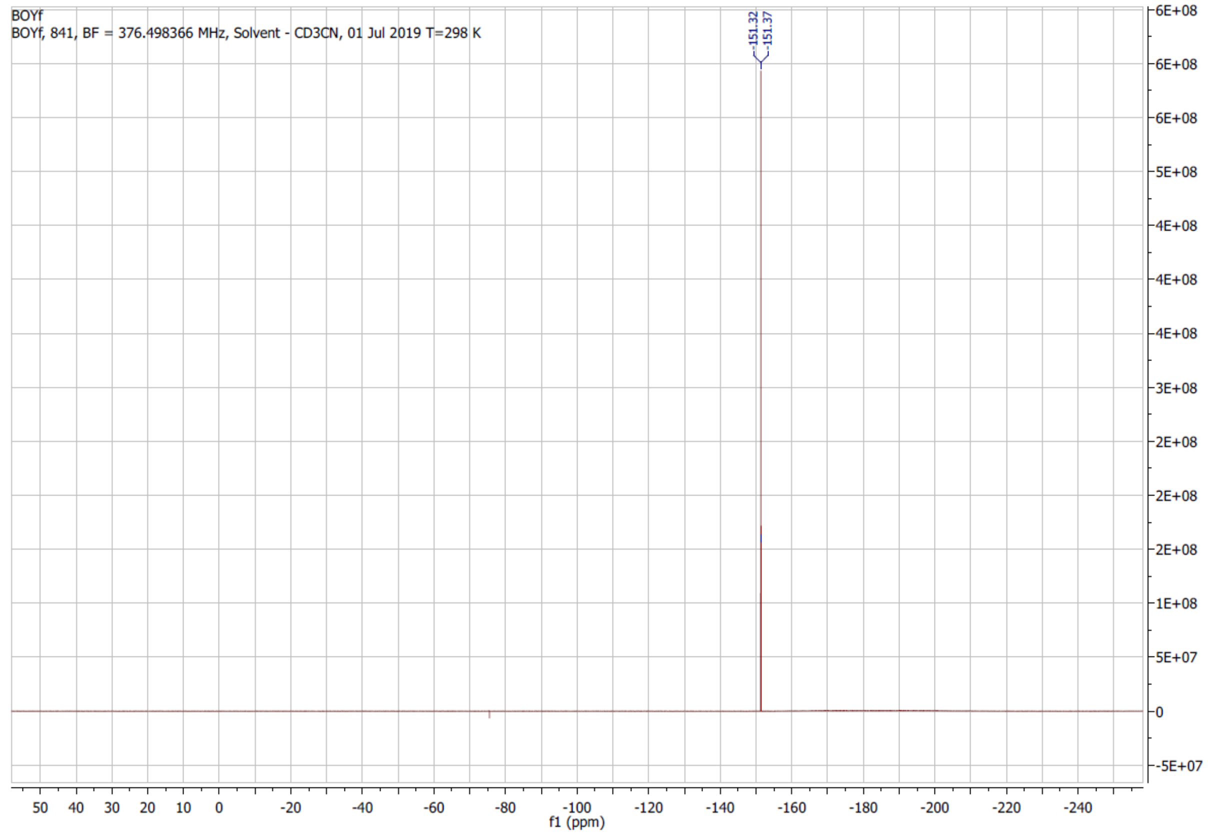
BOYf
BOYf, 841, BF = 376.498366 MHz, Solvent - CD3CN, 01 Jul 2019 T=298 K
-151.32
-151.37
f1 (ppm)

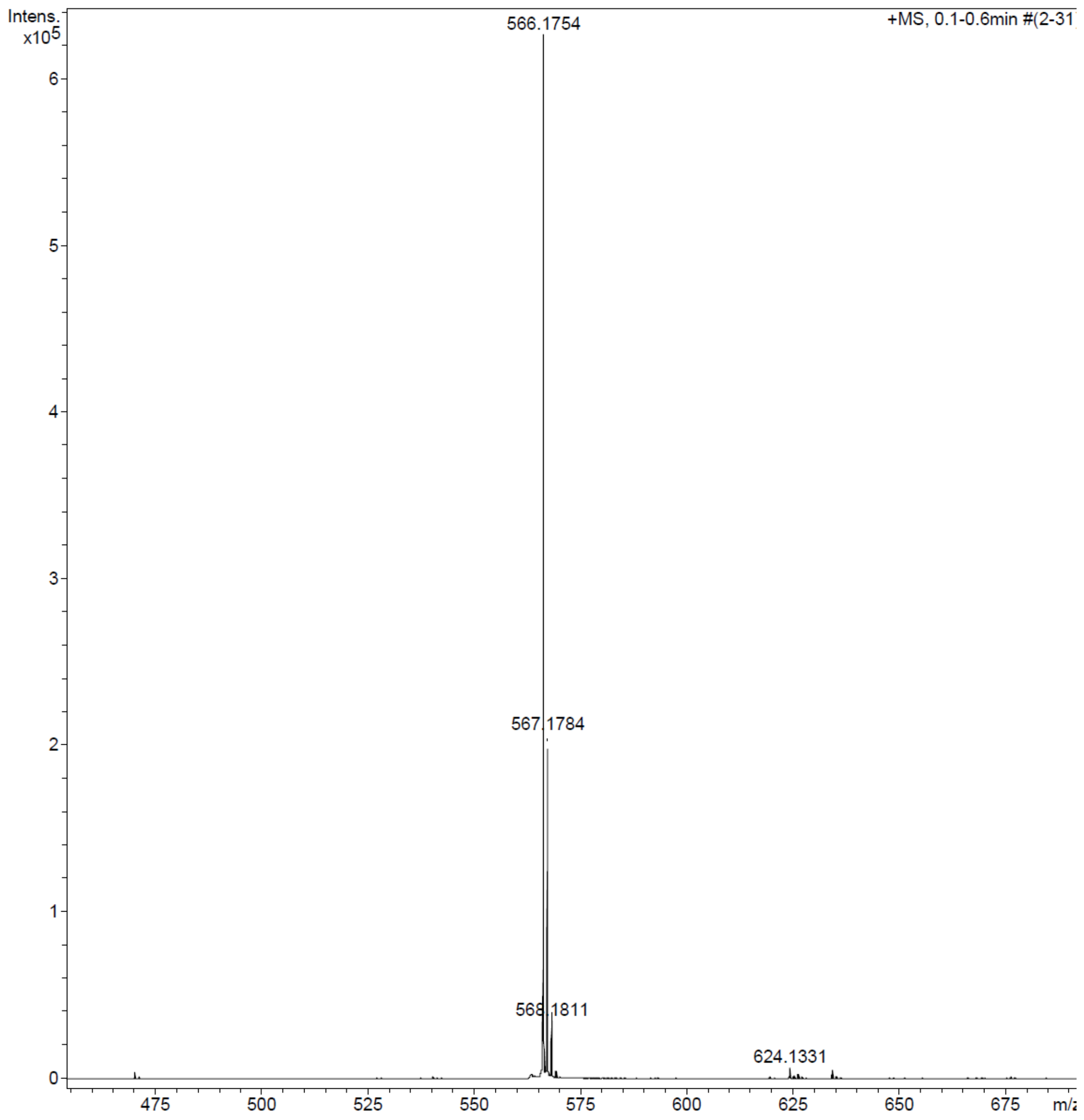

Intens.
x10^5
+MS, 0.1-0.6min #(2-31)
566.1754
567.1784
568.1811
624.1331
6
5
4
3
2
1
0
475
500
525
550
575
600
625
650
675
m/z

Chromatograms (TLC and HPLC)

4-[^{18}F]Fluoroanisole ([^{18}F]**2**):

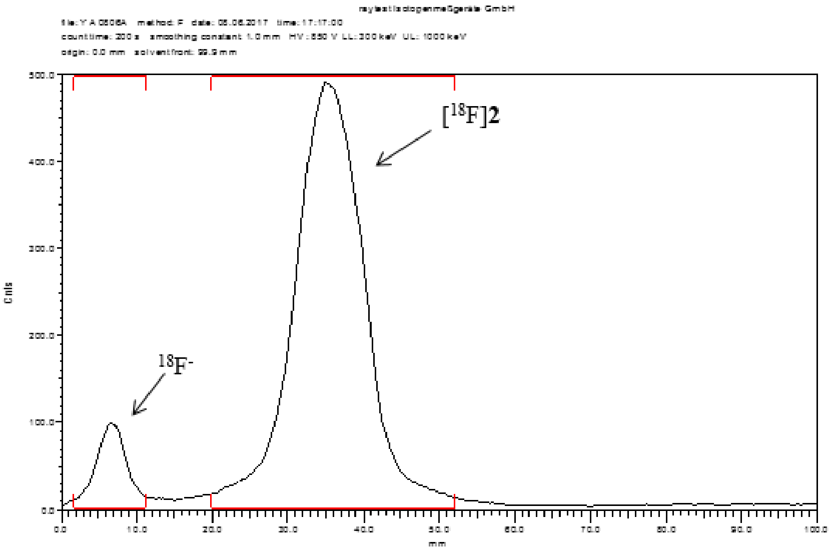

4-[^{18}F]Fluoroanisole, TLC in hexane/ethylacetate (1/4)

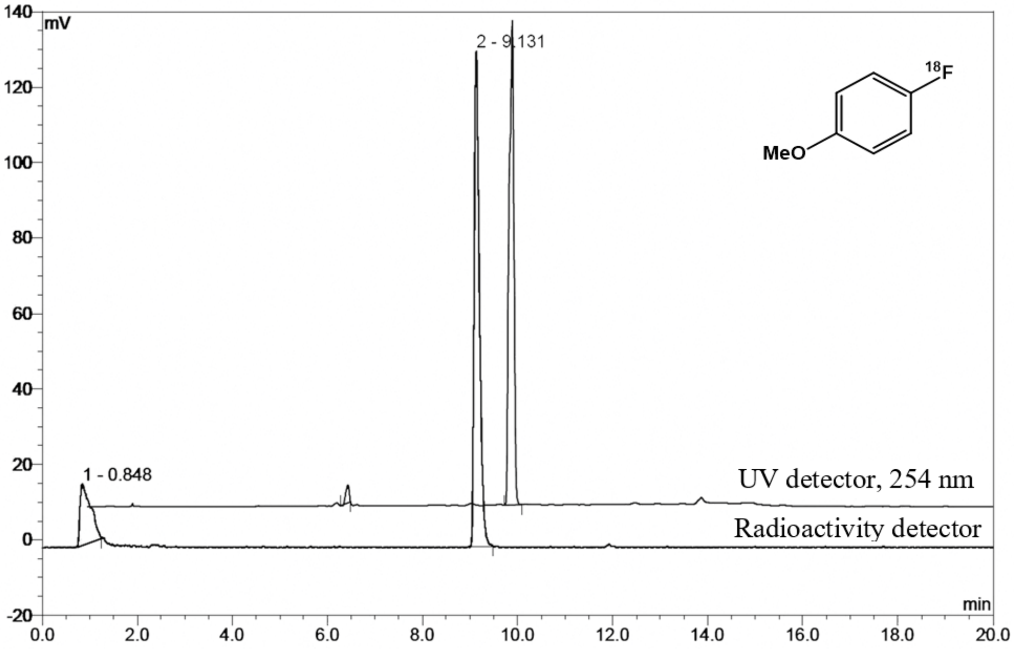

4-[^{18}F]Fluoroanisole and the reference compound, HPLC on X-Bridge C18 column 5 µm, 150×4.6 mm (Waters), 0–2 min: 5% MeCN, 2–13 min: 5→90% MeCN (2.0 mL/min), injection loop: 20 µL.

[^{18}F]Fluorobenzene ([^{18}F]**3**):

TLC was not applicable for [^{18}F]fluorobenzene because of volatility of the substance

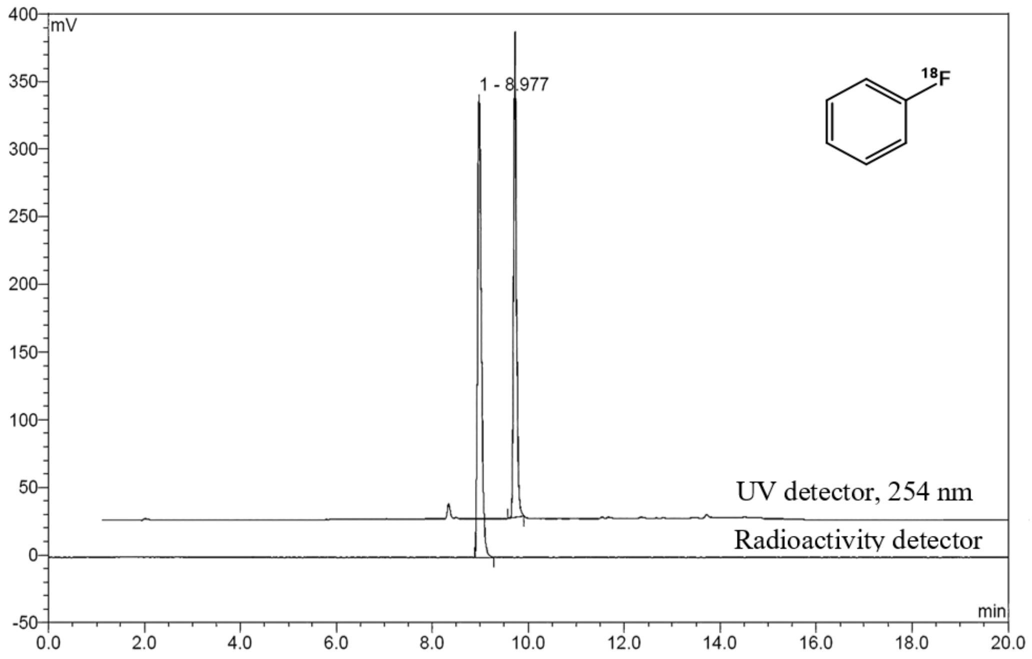

[^{18}F]Fluorobenzene and the reference compound, HPLC on X-Bridge C18 column 5 µm, 150×4.6 mm (Waters), 0–2 min: 5% MeCN, 2–13 min: 5→90% MeCN (2.0 mL/min), injection loop: 20 µL.

3-[^{18}F]Fluorobenzaldehyde ([^{18}F]**4**):

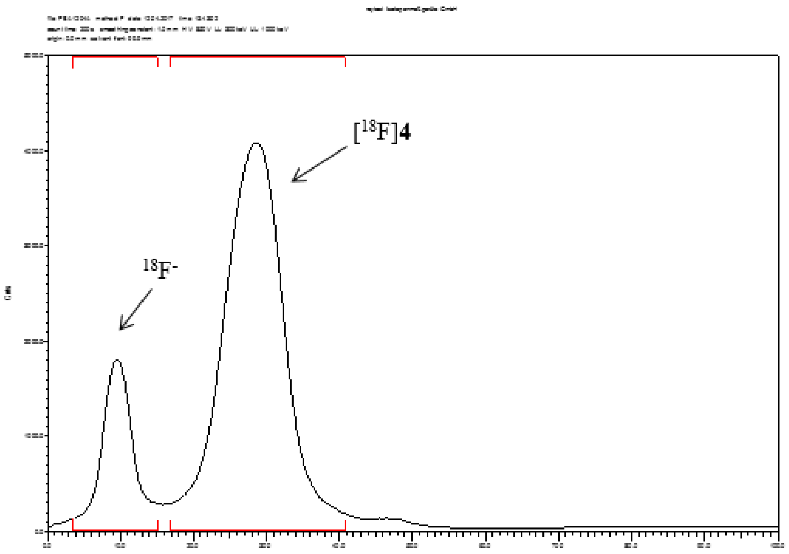

3-[^{18}F]Fluorobenzaldehyde ([^{18}F]**4**), TLC in hexane/ethylacetate (1/4)

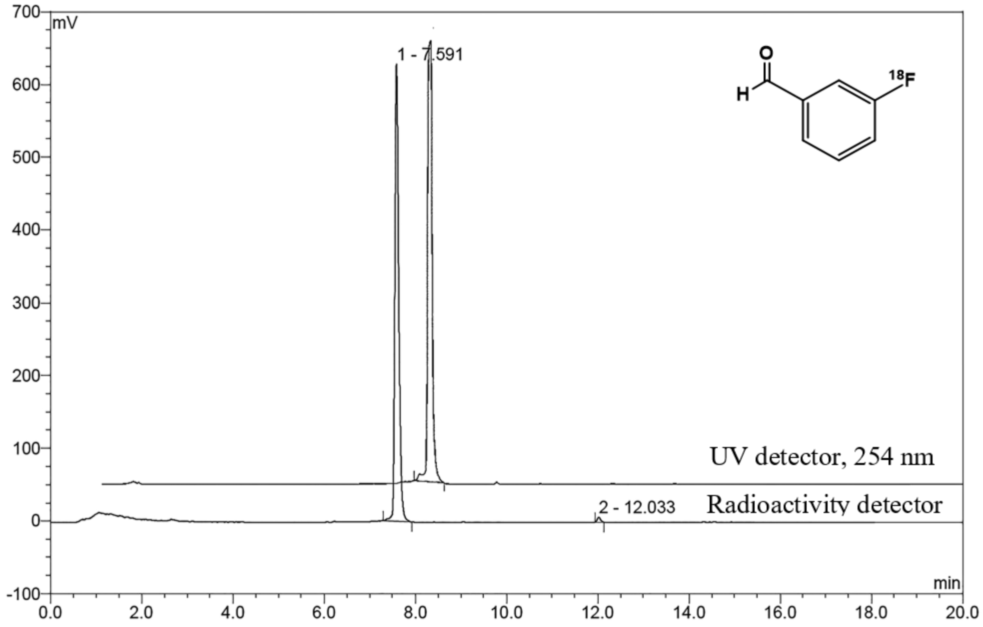

3-[^{18}F]Fluorobenzaldehyde ([^{18}F]**4**) and the reference compound, HPLC on X-Bridge C18 column 5 µm, 150×4.6 mm (Waters), 0–2 min: 5% MeCN, 2–13 min: 5→90% MeCN (2.0 mL/min), UV detection: 254 nm, injection loop: 20 µL.

Compound	k	R_t
[^{18}F]**2**	9.8	00:09:06
[^{18}F]**3**	9.6	00:08:52
[^{18}F]**4**	8.0	00:07:36
Dead time		00:00:50

Reference compounds: Phe, 2- and 4-FPhe:

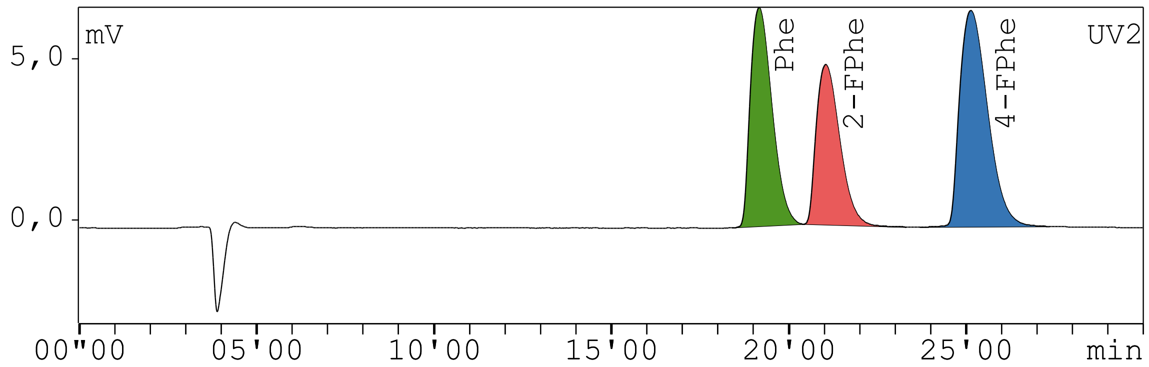

Synergy Hydro-RP (4.6×250 mm), 6% EtOH (1.0 mL/min), 210 nm, 20 μL loop

Compound	k'	R_t
**Phe**	4.6	00:19:19
**2-FPhe**	5.1	00:21:02
**4-FPhe**	6.3	00:25:08
Dead time		00:03:28

Boc$_2$-2-[^{18}F]FPhe-O*t*Bu ([^{18}F]**5a**) and Boc-2-[^{18}F]FPhe-O*t*Bu ([^{18}F]**5b**):

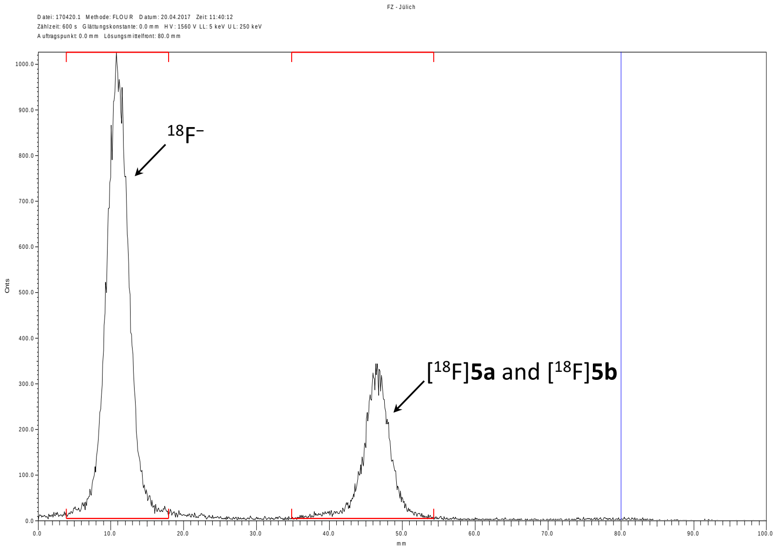

TLC in hexane/ethylacetate (9/1)

2-[^{18}F]FPhe after HPLC purification: Quality control:

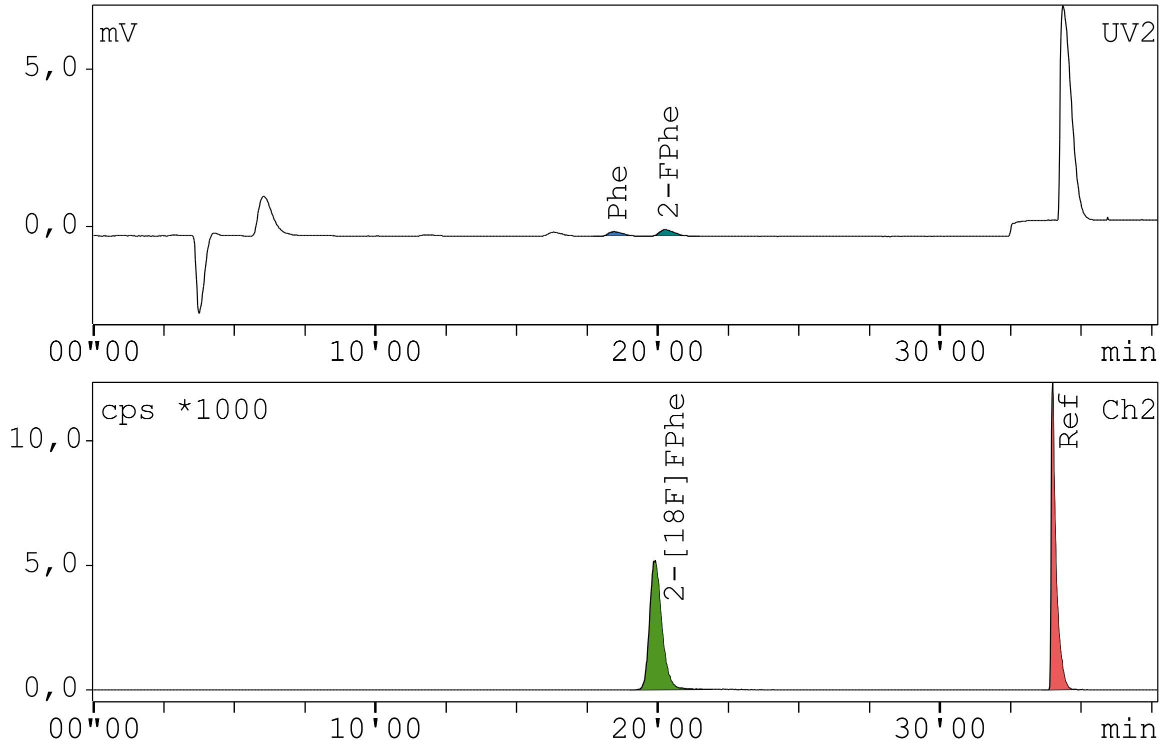

Synergy Hydro-RP (4.6×250 mm), 6% EtOH (1.0 mL/min), 210 nm, 20 µL loop.

2-[^{18}F]FPhe after HPLC purification: Spiking with the reference compound:

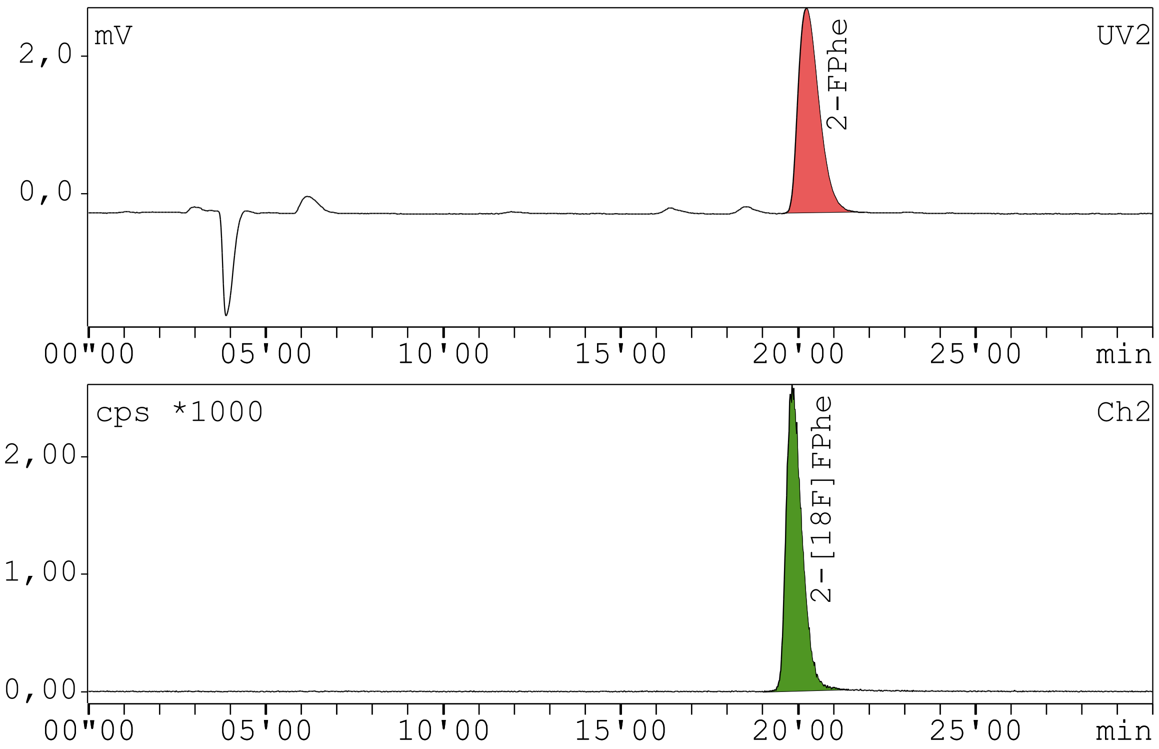

Synergy Hydro-RP (4.6×250 mm), 6% EtOH (1.0 mL/min), 210 nm, 20 µL loop.

Reference compounds: (*S*)- and (*R*)-2-FPhes:

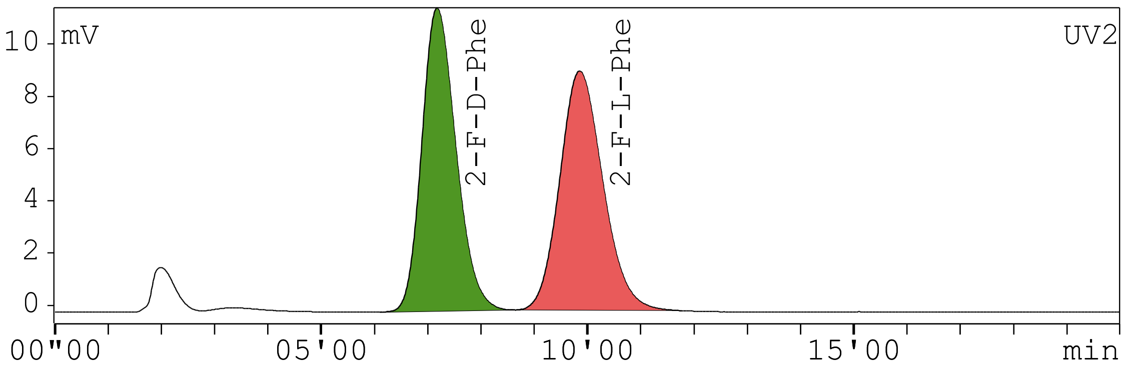

Crownpak (+) (4.6×150 mm), 0.025 M $HClO_4$ (1.0 mL/min), 210 nm, 20 µL loop.

Compound	K'	R_t
(*R*)-2-FPhe	2.5	00:07:11
(*S*)-2-FPhe	3.7	00:09:51
Dead time		00:02:05

2-[^{18}F]FPhe after HPLC purification: Determination of enantiomeric purity, quality control:

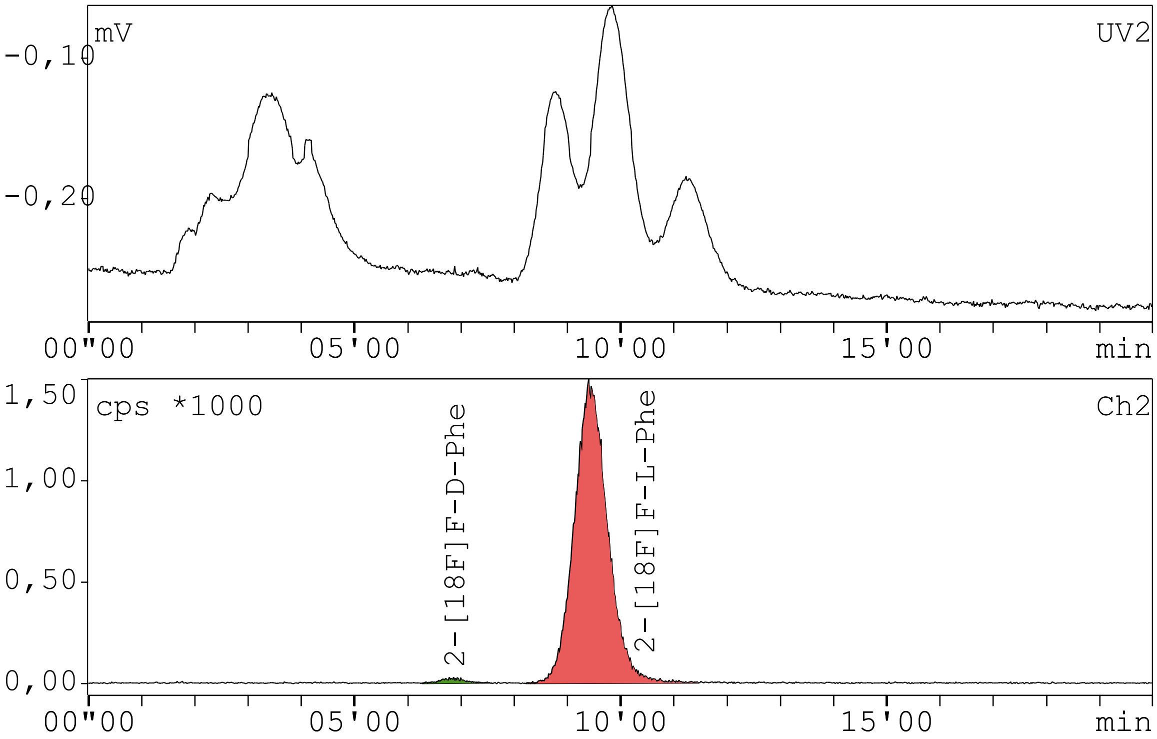

Crownpak (+) (4.6×150 mm), 0.025 M $HClO_4$ (1.0 mL/min), 210 nm, 20 µL loop.

2-[^{18}F]FPhe after HPLC purification: Determination of enantiomeric purity, spiking with (*S*)- and (*R*)-2-FPhes:

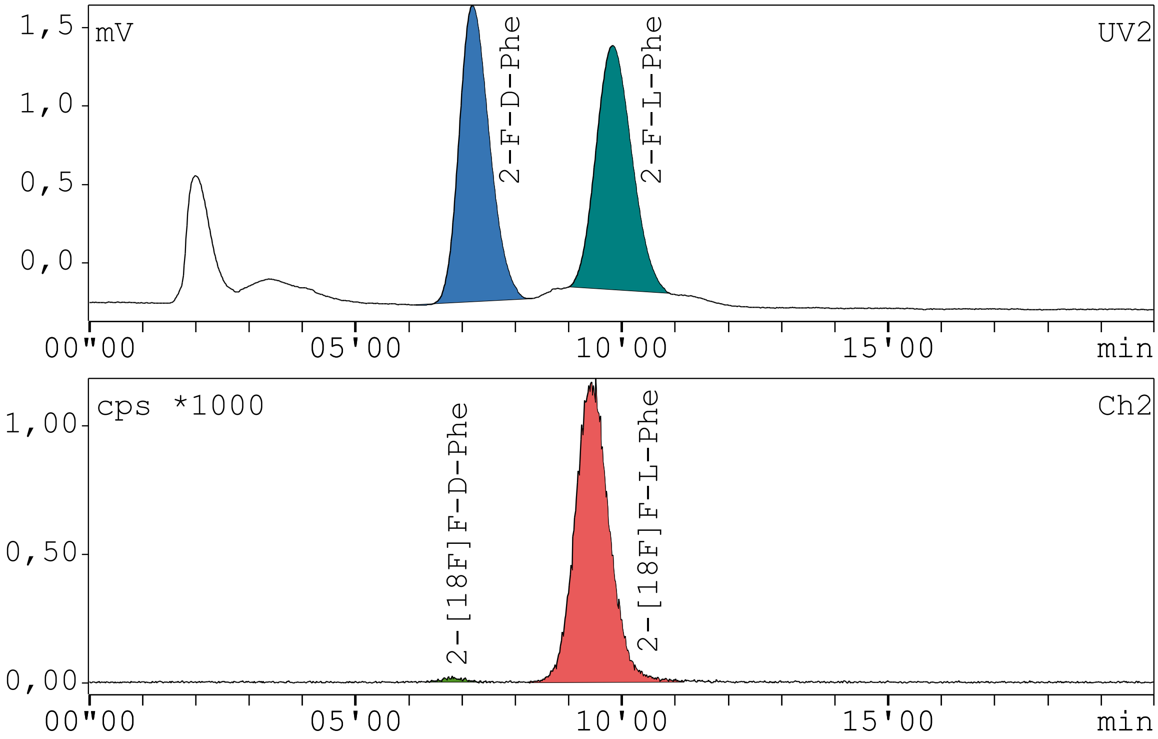

Crownpak (+) (4.6×150 mm), 0.025 M $HClO_4$ (1.0 mL/min), 210 nm, 20 µL loop.

Boc-3-[^{18}F]FPhe-O*t*Bu ([^{18}F]**6**) (TLC):

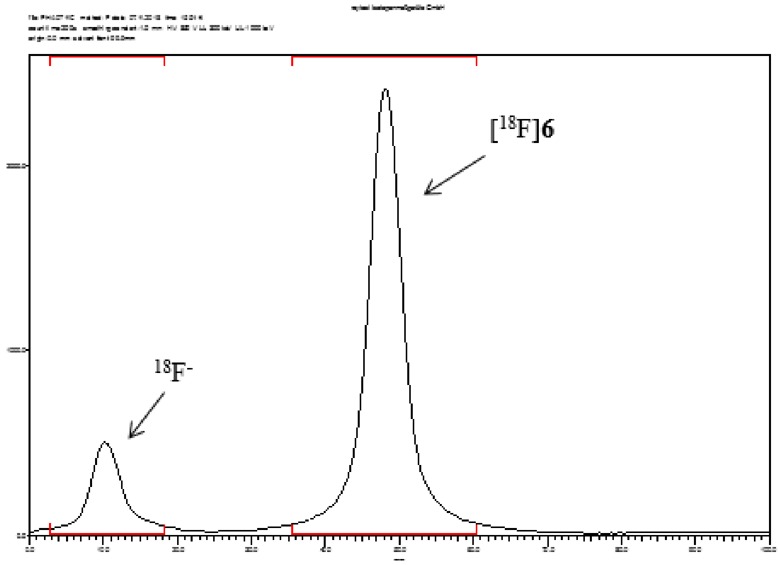

EtOAc/$CHCl_3$/AcOH (4/1/1)

Boc-3-[^{18}F]FPhe-O*t*Bu ([^{18}F]**6**) (HPLC):

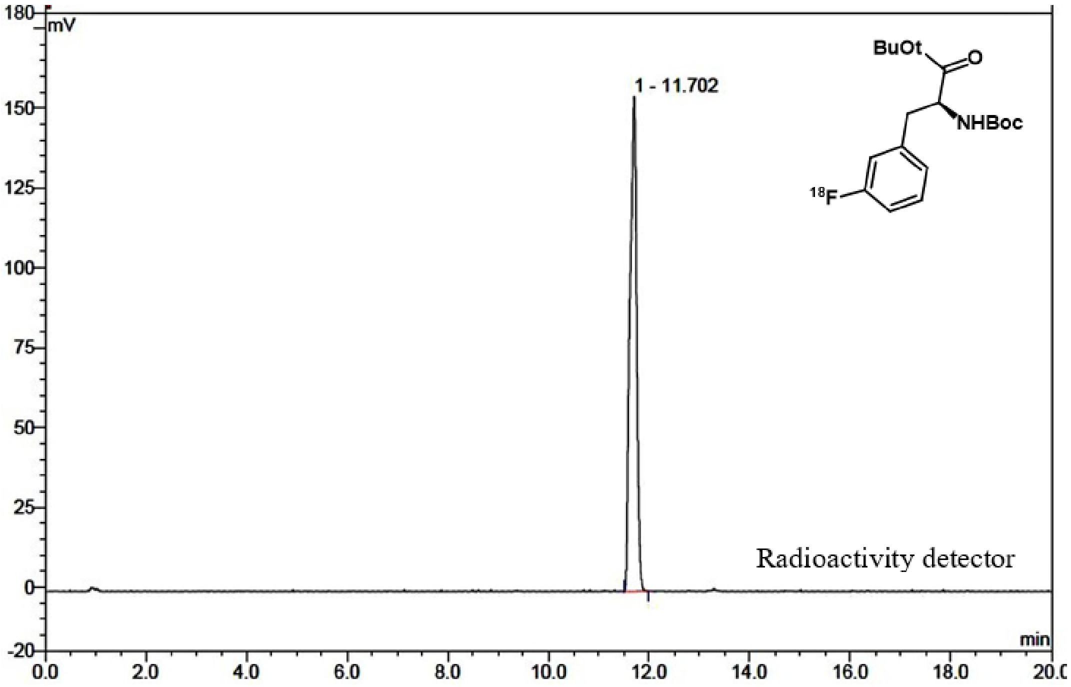

Boc-3-[^{18}F]FPhe-O*t*Bu ([^{18}F]**6**), X-Bridge C18 column 5 μm, 150×4.6 mm (Waters), 0–2 min: 5% MeCN, 2–13 min: 5→90% MeCN (2.0 mL/min).

Compound	k'	R_t
[^{18}F]**6**	13.5	00:11:40
Dead time		00:00:50

3-[^{18}F]FPhe after HPLC purification: Quality control. Spiking with the reference compound:

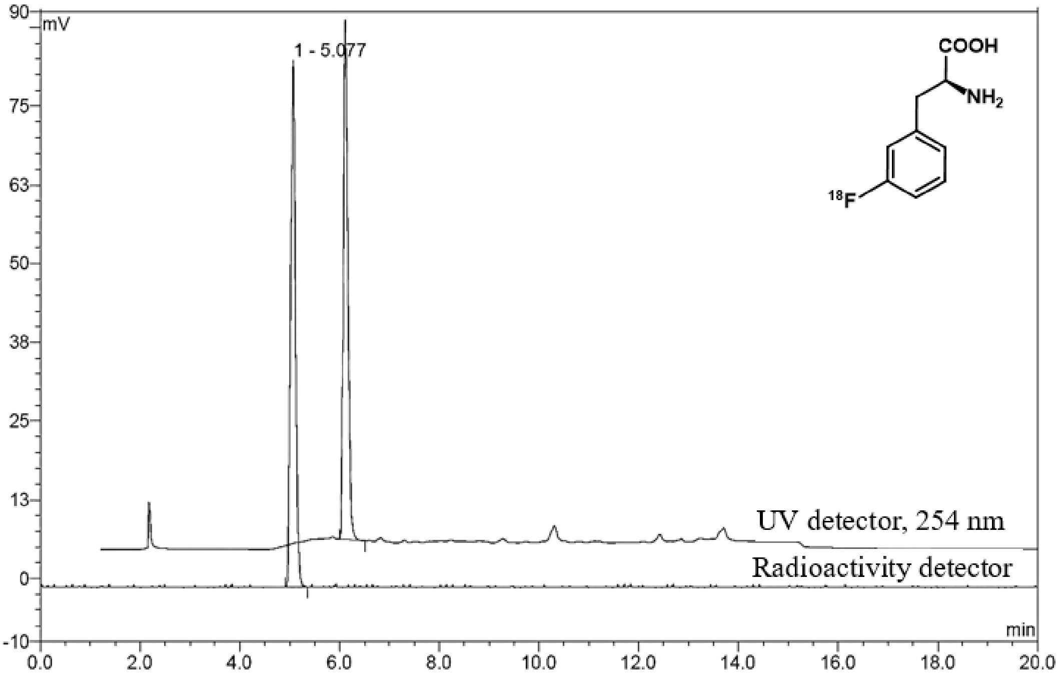

(HPLC):

3-[^{18}F]FPhe and the reference compound, X-Bridge C18 column 5 µm, 150×4.6 mm (Waters), 0–2 min: 5% MeCN, 2–13 min: 5→90% MeCN (2.0 mL/min), UV detection: 254 nm, injection loop: 20 µL.

3-[^{18}F]FPhe after HPLC purification: Determination of enantiomeric purity, spiking with (*S*)- and (*R*)-3-FPhes:

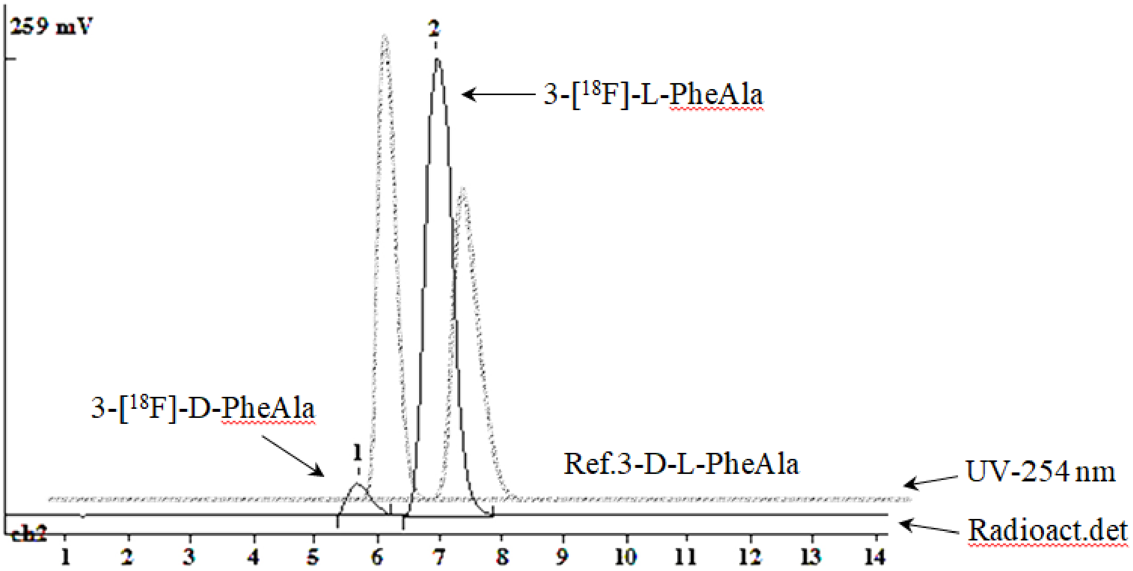

Crownpak (+) (4.6×150 mm) (Daicel) column, an aqueous 10% MeOH in $HClO_4$ (pH 2) (0.8 mL/min), 254 nm, 20 µL loop; 94.7% purity.

Boc$_2$-4-[^{18}F]FPhe-O*t*Bu ([^{18}F]**7a**) and Boc-4-[^{18}F]FPhe-O*t*Bu ([^{18}F]**7b**) (TLC):

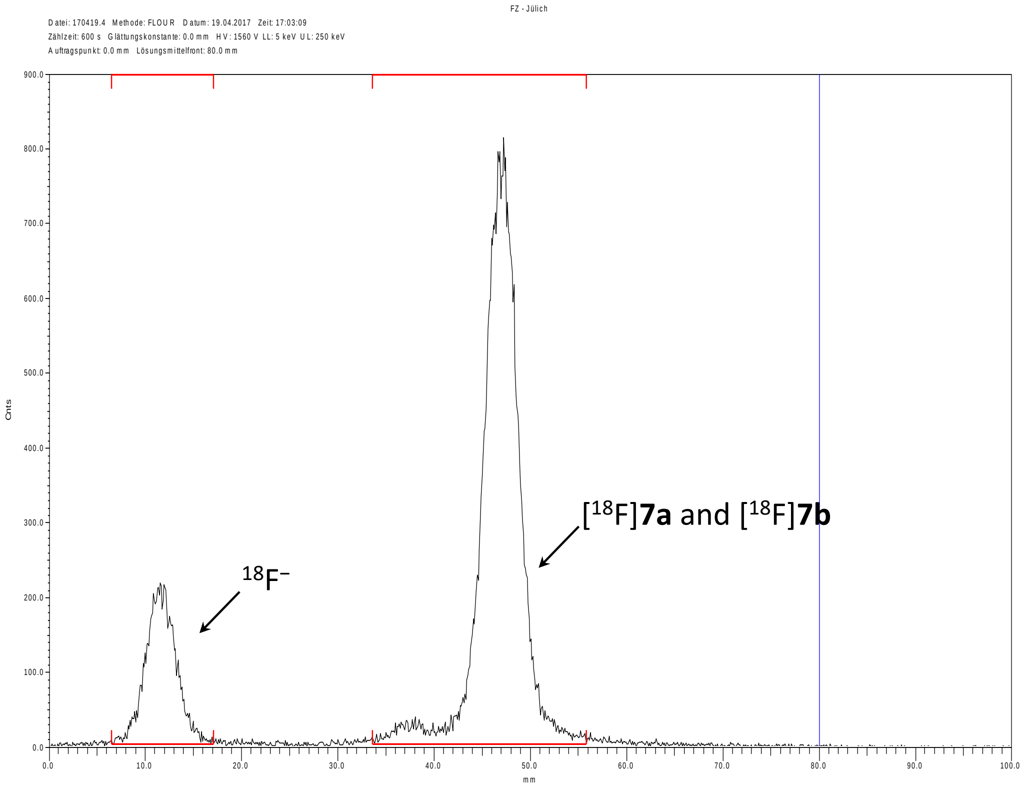

Boc$_2$-4-[^{18}F]FPhe-O*t*Bu ([^{18}F]**7a**) (HPLC):

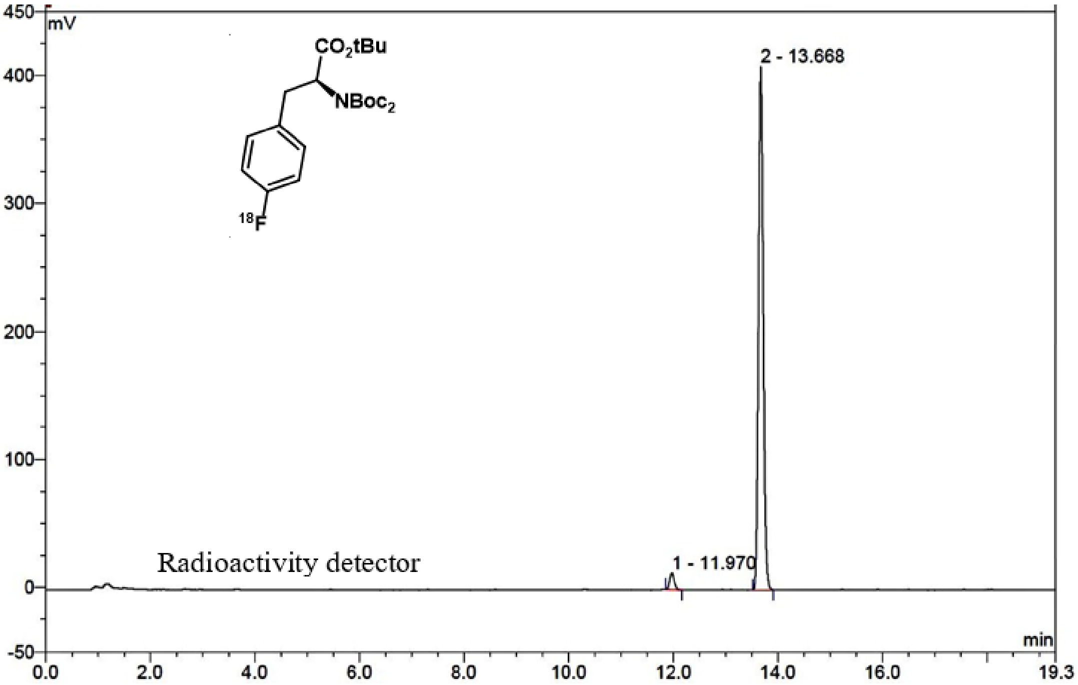

Boc$_2$-4-[^{18}F]FPhe-O*t*Bu ([^{18}F]**7a**), X-Bridge C18 column 5 µm, 150 × 4.6 mm (Waters), 0–2 min: 5% MeCN, 2–13 min: 5→90% MeCN (2.0 mL/min).

Boc-4-[^{18}F]FPhe-O*t*Bu ([^{18}F]**7b**) (HPLC):

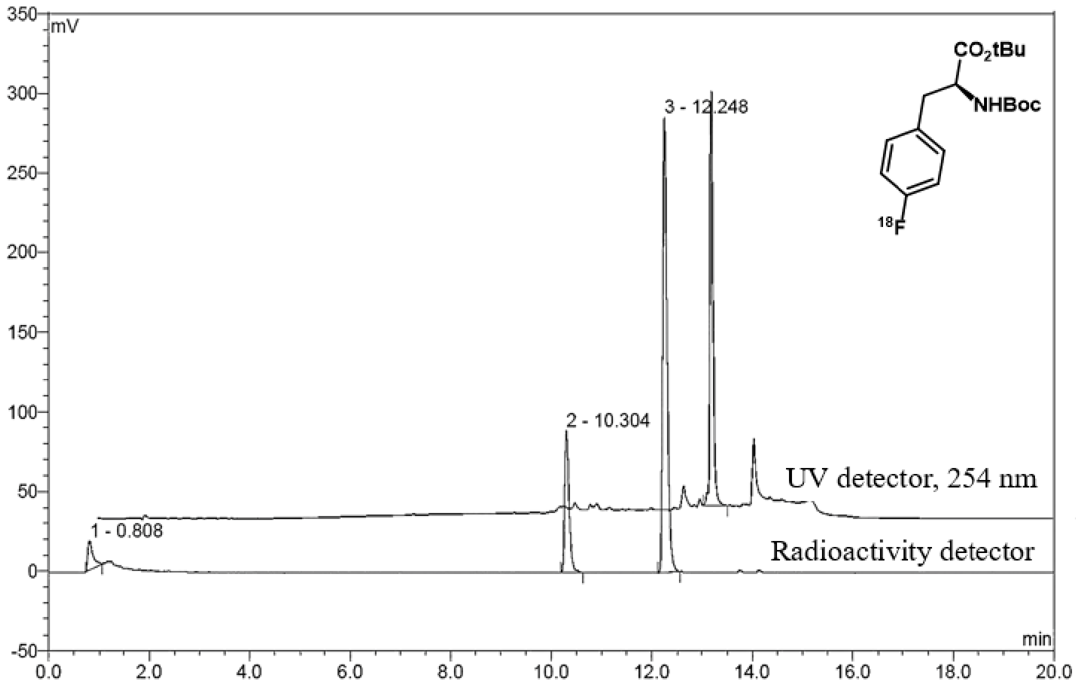

Boc-4-[^{18}F]FPhe-O*t*Bu ([^{18}F]**7b**) and the reference compound, HPLC on X-Bridge C18 column 5 µm, 150×4.6 mm (Waters), 0–2 min: 5% MeCN, 2–13 min: 5→90% MeCN (2.0 mL/min), UV detection: 254 nm, injection loop: 20 µL.

Compound	k'	R_t
[^{18}F]**7a**	16.1	00:13:42
[^{18}F]**7b**	14.3	00:12:12
Dead time		00:00:50

4-[^{18}F]FPhe after HPLC purification: Quality control:

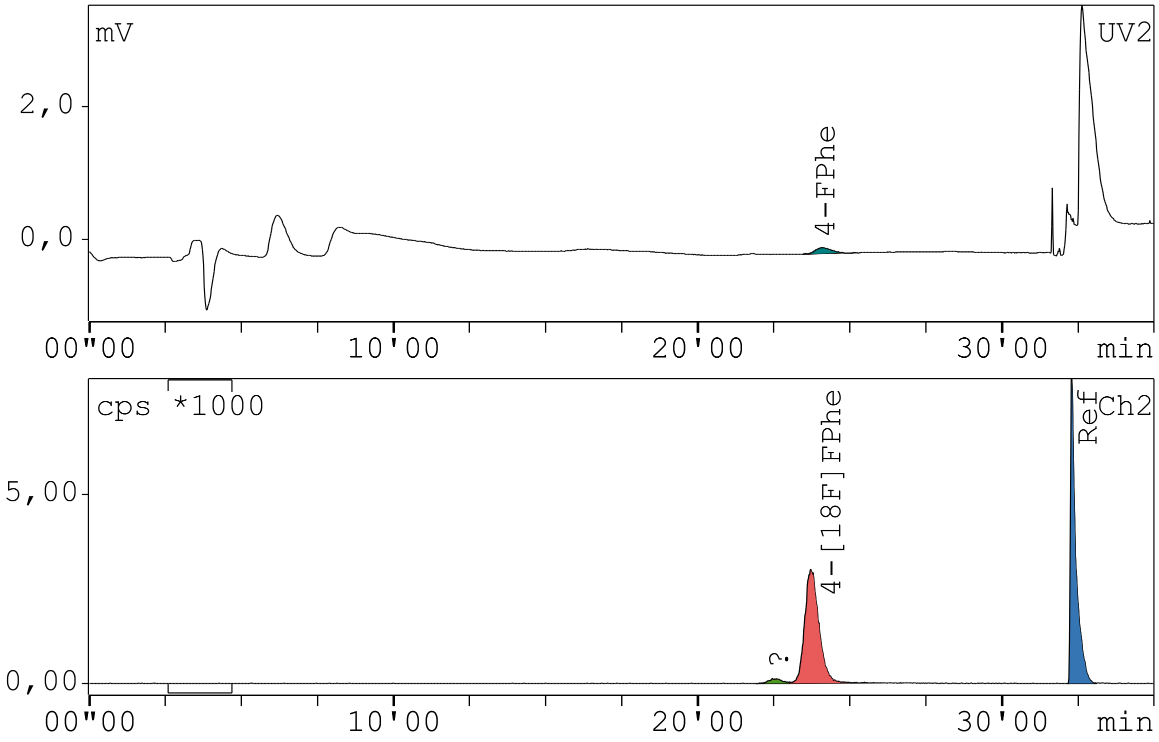

Synergy Hydro-RP (4.6×250 mm), 6% EtOH (1.0 mL/min), 210 nm, 20 µL loop.

4-[^{18}F]FPhe after HPLC purification: Spiking with the reference compound:

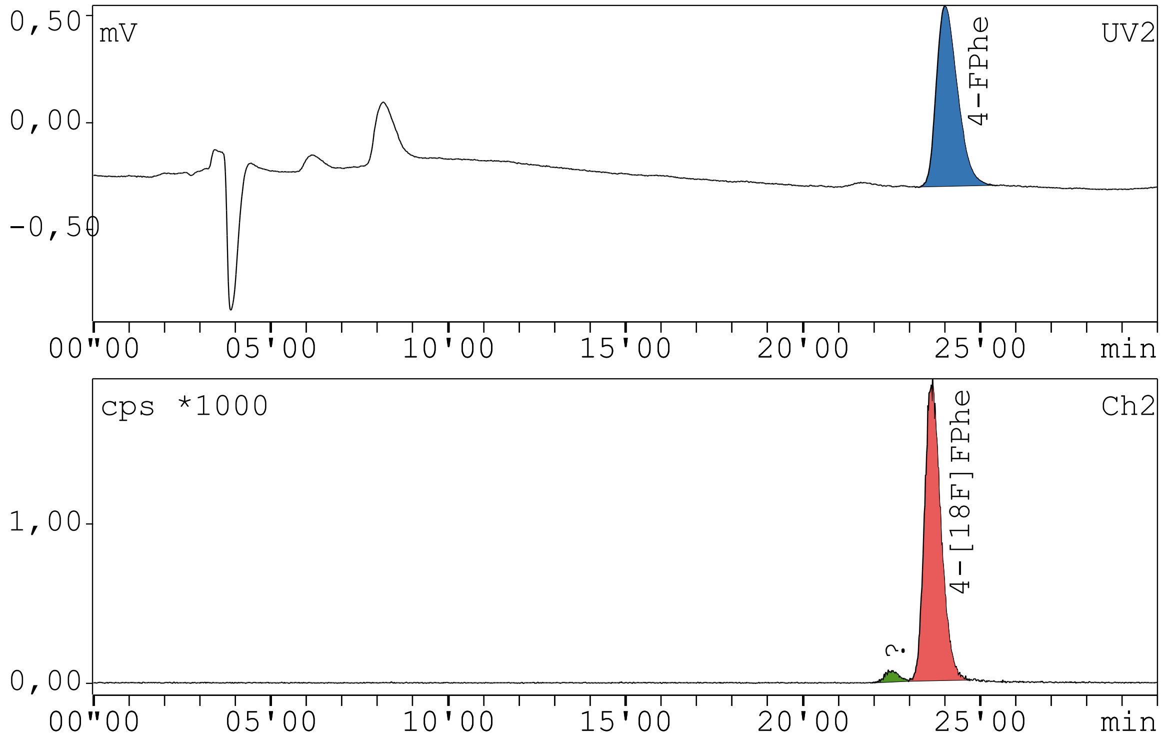

Synergy Hydro-RP (4.6×250 mm), 6% EtOH (1.0 mL/min), 210 nm, 20 µL loop.

Reference compounds: (*S*)- and (*R*)-4-FPhes:

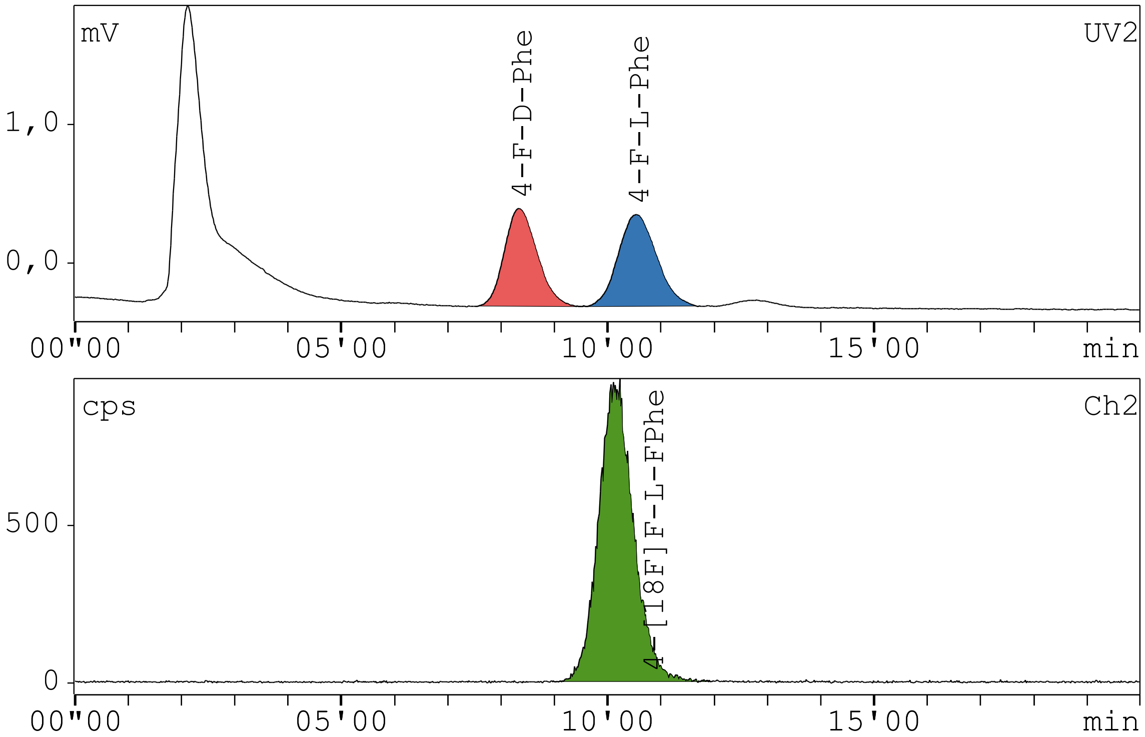

Crownpak (+) (4.6×150 mm), 0.025 M $HClO_4$ (1.0 mL/min), 210 nm, 20 μL loop.

Compound	K'	R_t
(*R*)-4-FPhe	3.0	00:08:16
(*S*)-4-FPhe	4.1	00:10:30
Dead time		00:02:05

4-[^{18}F]FPhe after HPLC purification: Determination of enantiomeric purity, quality control:

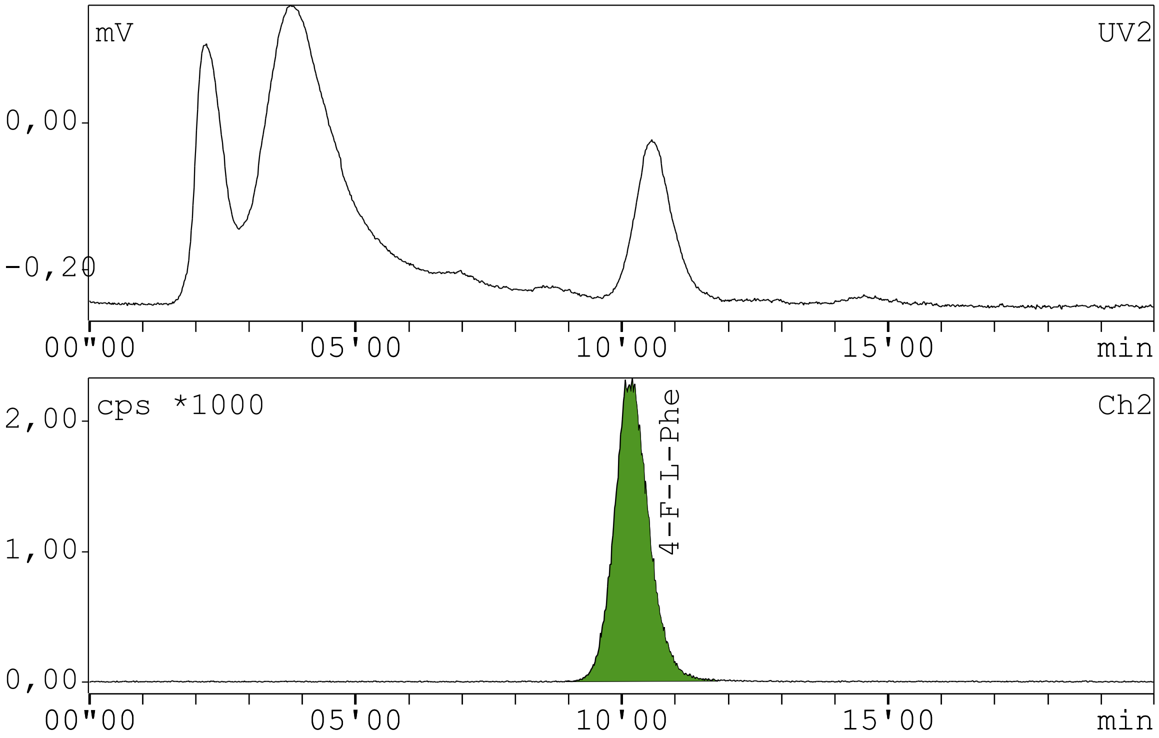

Crownpak (+) (4.6×150 mm), 0.025 M $HClO_4$ (1.0 mL/min), 210 nm, 20 µL loop.

4-[^{18}F]FPhe after HPLC purification: Determination of enantiomeric purity, spiking with (*S*)- and (*R*)-4-FPhes:

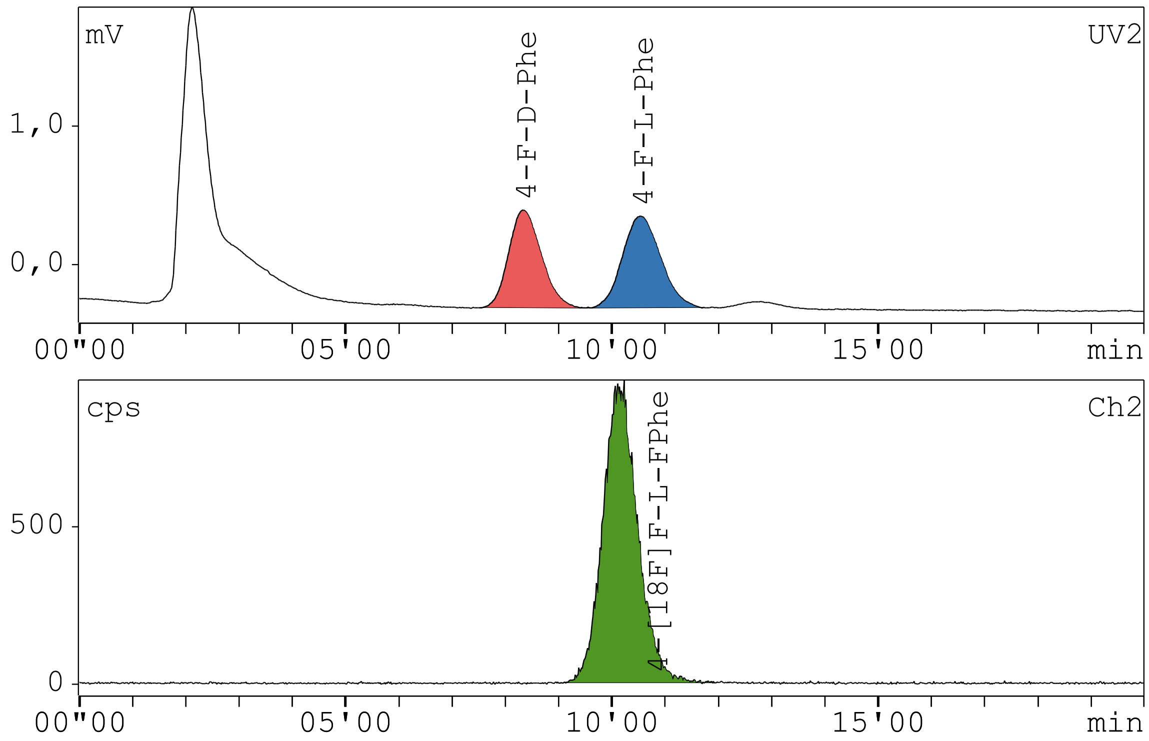

Crownpak (+) (4.6×150 mm), 0.025 M $HClO_4$ (1.0 mL/min), 210 nm, 20 µL loop.
